# HSP70-Homolog DnaK of *Pseudomonas aeruginosa* Increases the Production of IL-27 through Expression of *EBI3* via TLR4-Dependent NF-κB and TLR4-Independent Akt Signaling

**DOI:** 10.3390/ijms21239194

**Published:** 2020-12-02

**Authors:** Jisu Jeon, Yeji Lee, Hyeonseung Yu, Un-Hwan Ha

**Affiliations:** Department of Biotechnology and Bioinformatics, Korea University, Sejong 30019, Korea; allonso@korea.ac.kr (J.J.); yejee90@korea.ac.kr (Y.L.); cockychild@korea.ac.kr (H.Y.)

**Keywords:** Akt, DnaK, EBI3, IL-27, NF-κB, *Pseudomonas aeruginosa*, TLR4

## Abstract

IL-27, a heterodimeric cytokine composed of the p28 subunit and Epstein–Barr virus-induced gene 3 (EBI3), acts as a potent immunosuppressant and thus limits pathogenic inflammatory responses. IL-27 is upregulated upon *Pseudomonas aeruginosa* infection in septic mice, increasing susceptibility to the infection and decreasing clearance of the pathogen. However, it remains unclear which *P. aeruginosa*-derived molecules promote production of IL-27. In this study, we explored the mechanism by which *P. aeruginosa* DnaK, a heat shock protein 70-like protein, induces *EBI3* expression, thereby promoting production of IL-27. Upregulation of *EBI3* expression did not lead to an increase in IL-35, which consists of the p35 subunit and EBI3. The IL-27 production in response to DnaK was biologically active, as reflected by stimulation of IL-10 production. DnaK-mediated expression of *EBI3* was driven by two distinct signaling pathways, NF-κB and Akt. However, NF-κB is linked to TLR4-associated signaling pathways, whereas Akt is not. Taken together, our results reveal that *P. aeruginosa* DnaK potently upregulates *EBI3* expression, which in turn drives production of the prominent anti-inflammatory cytokine IL-27, as a consequence of TLR4-dependent activation of NF-κB and TLR4-independent activation of the Akt signaling pathway.

## 1. Introduction

*Pseudomonas aeruginosa* is an opportunistic bacterial pathogen that causes localized infections at various sites in the body, including the respiratory tract, and can spread systemically, especially in immunocompromised patients [1,2]. Upon infection of the respiratory tract, *P. aeruginosa* encounters epithelial cells lining the host airways and patrolling macrophages, which represent the first lines of host defense [3]. These cells stimulate inflammatory responses against infection by inducing the expression of proinflammatory cytokines, resulting in recruitment of neutrophils to the site of infection [4]. However, balanced expression of anti-inflammatory cytokines is critical for effective immunity [5], which requires for not only effective clearance of invading pathogens but also avoidance of tissue damage [6]. Microbial pathogens have evolved many ingenious ways to evade host defense responses and phagocytic clearance [7,8]. One mechanism that pathogens used to disrupt host defenses is modulation of cytokine expression—in particular, the upregulation of the production of anti-inflammatory cytokines.

Interleukin-27 (IL-27), which acts as a potent immunosuppressant, consists of an alpha subunit (IL-27 p28) and Epstein–Barr virus-induced gene 3 (EBI3) [9,10]. It is expressed mainly by myeloid cells, such as macrophages and dendritic cells [11]. IL-27 regulates the biological functions of cells by driving production of IL-10, a key anti-inflammatory cytokine [12]. Elevated levels of IL-10 increase susceptibility to secondary nosocomial infections in sepsis [13]. Consistent with this, a virulence factor of *Bordetella pertussis* upregulates expression of IL-10 in macrophages, thereby suppressing the development of cell-mediated immunity and limiting the clearance of invading pathogens [14]. Therefore, reduction in the IL-27 level promotes bacterial clearance, improves host response, and decreases mortality [15]. The plasma concentration of IL-27 is significantly elevated in murine models of sepsis, as well as in human patients with this condition; accordingly, it is considered to be a potential diagnostic marker for sepsis [16,17]. Following sepsis and secondary intrapulmonary challenges with *P. aeruginosa*, neutralization of IL-27 significantly improves survival of septic mice and clearance of *P. aeruginosa*; conversely, direct application of recombinant IL-27 increases susceptibility to *P. aeruginosa* infection [16]. Given that the only available treatment options for combating bacterial sepsis are antibiotics and supportive care [18], IL-27 represents a promising therapeutic target.

The molecular chaperones known as heat shock proteins (HSPs) are highly conserved in all domains of life and play important roles in housekeeping functions, including assembly and disassembly of protein complexes, and folding and unfolding of proteins [19]. Eukaryotic HSPs have a wide range of cytoprotective functions; they are expressed in response to environmental stresses, including infection, and can modulate the host immune system [20]. Similar to eukaryotic HSPs, bacterial HSPs can also modulate innate and adaptive immune responses [21,22,23,24]. Bacterial HSPs have been classified into several groups according to their molecular weight. Well-characterized HSPs include DnaK/DnaJ/GrpE and GroEL/GroES, which play essential roles in many physiological processes [25,26]. As intracellular chaperones, these HSPs mainly function to prevent synthesized proteins from aggregating into nonfunctional forms. In order to provide the function of chaperon, they are mainly located in intracellular space. However, it was shown that DnaK is also located in periplasmic space [27] and even secreted extracellularly [28], although the method of secretion is not known. Consequently, DnaK could act as a cytosolic, periplasmic and extracellular proteins to play diverse physiological and pathological roles.

Intracellular mammalian HSP70 plays a role in regulating inflammation, specifically via the NLRP3 inflammasome in a Nuclear Factor-κB (NF-κB)-independent manner [29]. However, it was shown that extracellular HSP70, which functions as a damage-associated molecular pattern (DAMP) released from damaged cells under necrotic conditions, regulates inflammatory responses, thus leading to NF-κB activation [30] as well as enhancing cytolytic capacities of natural killer cells [31]. These results provide the diverse roles of HSP70 both inside and outside of mammalian cells. Similarly, as pathogen-associated molecular patterns (PAMPs) released by *P. aeruginosa*, DnaK is a highly conserved protein similar to mammalian HSP70 protein. In human macrophages, DnaK moderately triggers the induction of IL-1β production via activation of both NF-κB and c-Jun N-terminal kinase (JNK) signaling pathways [32]. However, there is a negative regulation of DnaK-mediated IL-1β expression via cross-talk between JNK and the activation of the phosphoinositide 3-kinase signaling pathways [33], implying diverse immune regulation in response to DnaK.

In this study, we sought to understand the inflammatory roles of the HSP70 homolog DnaK. Eukaryotic HSP70 can both activate and suppress immune responses [30,34,35,36,37]. However, the detailed mechanisms by which *P. aeruginosa* DnaK suppresses immune responses have not been fully elucidated. Here, we demonstrate that DnaK induces *EBI3* expression, thereby increasing production of the anti-inflammatory cytokine IL-27. This induction is dependent on activation of the NF-κB and Akt signaling pathways. However, NF-κB activation is under the control of toll-like receptor 4 (TLR4), whereas Akt activation is not. Our results provide new insights into the roles of DnaK in the induction of anti-inflammatory cytokine expression during host–*Pseudomonas* interactions.

## 2. Results

### 2.1. P. aeruginosa-Mediated Induction of EBI3 Expression

**Figure 1:**
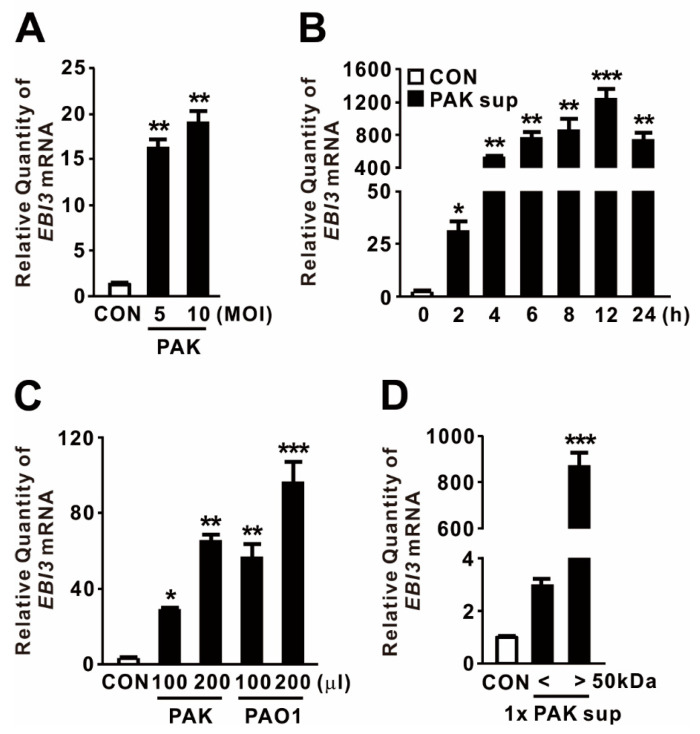
*P. aeruginosa*-mediated induction of Epstein–Barr virus-induced gene 3 (*EBI3)* expression. (**A**) dTHP-1 cells were treated with *P. aeruginosa* strain PAK at a multiplicity of infection (MOI) of 5 or 10 for 4 h. (**B**) dTHP-1 cells were treated for the indicated times with 100 μL culture supernatant (Sup) obtained from PAK. (**C**) dTHP-1 cells were treated for 4 h with 100 or 200 μL Sup obtained from *P. aeruginosa* strains PAK and PAO1. (**D**) dTHP-1 cells were treated for 4 h with 100 μL Sup, size-fractionated using a 50 kDa pore size membrane filter. After treatment, *EBI3* mRNA levels were quantified by qRT-PCR. Data are expressed as means ±SD (*n* = 3). *, *p* < 0.05; **, *p* < 0.01; ***, *p* < 0.001 vs. CON. MOI, multiplicity of infection.

EBI3 was initially discovered in the supernatant of Epstein–Barr virus-infected B-cells, and its expression is triggered by stimuli such as mitogen activation [38]. To examine whether *P. aeruginosa* infection drives the expression of *EBI3*, dTHP-1 human macrophage cells were treated with *P. aeruginosa* strain PAK at a multiplicity of infection (MOI) of 5 or 10. As shown in Figure 1A, the treatment significantly elevated *EBI3* expression, implying that *P. aeruginosa* acts as a potent inducer of *EBI3*. Next, we tested whether supernatants obtained from stationary-phase cultures of PAK are potent to trigger *EBI3* expression. The expression was gradually increased in a time-dependent manner in response to the supernatants, reaching a maximum at 12 h (Figure 1B). To evaluate the generalizability of *P. aeruginosa*-mediated *EBI3* expression, dTHP-1 cells were treated with supernatants obtained from cultures of *P. aeruginosa* strains PAK and PAO1. Both supernatants tested potently induced *EBI3* expression, suggesting that the bacterial factors contributing to the expression are produced and released by both strains (Figure 1C). To determine the responsible factors in the culture supernatant, we fractionated the supernatant through a centrifugal filter with 50 kDa pore sizes, and then used filtrates to treat dTHP-1 cells. The treatment clearly induced *EBI3* expression, indicating that the filtrate contains contributing factors, which are larger than 50 kDa (Figure 1D). Taken together, these results indicate that *EBI3* expression is potently induced by *P. aeruginosa* in macrophages and that the contributing factors are released into the culture supernatant.

### 2.2. P. aeruginosa DnaK Induces Expression of EBI3

**Figure 2:**
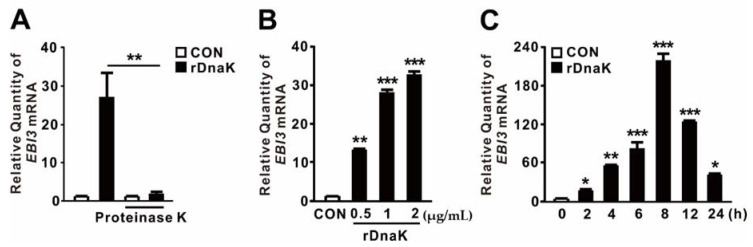
*P. aeruginosa*-derived DnaK induces expression of *EBI3*. (**A**) dTHP-1 cells were treated for 4 h with recombinant *P. aeruginosa* DnaK (rDnaK; 0.5 μg/mL) pretreated with Triton X-114. Treatment with proteinase K (20 μg/mL) was performed for 1 h. (**B**,**C**) dTHP-1 cells were treated with the indicated concentrations of rDnaK for 4 h (**B**) and 0.5 μg/mL rDnaK for the indicated times (**C**). After treatment, *EBI3* mRNA levels were quantified by qRT-PCR. Data are expressed as means ± SD (*n* = 3). *, *p* < 0.05; **, *p* < 0.01; ***, *p* < 0.001 vs. no treatment with proteinase K (**A**), CON (**B**,**C**).

HSPs act as endogenous DAMPs to transmit a “danger signal” that modulates secretion of inflammatory cytokines [39,40]. Recently, we showed that expression of the *dnaK* gene, which encodes an HSP70-like protein, is induced in *P. aeruginosa* upon infection of dTHP-1 cells [32]. Because DnaK has a molecular weight of ~79 kDa, we asked whether it contributes to the induction of *EBI3* expression. To determine the role of *P. aeruginosa* DnaK, we obtained Triton X-114-pretreated recombinant DnaK (rDnaK) as described previously [32,33]. As shown in Figure 2A, rDnaK treatment clearly induced *EBI3* expression, whereas pretreatment with proteinase K prevented induction, suggesting a role for DnaK. Induction of *EBI3* expression was dependent on rDnaK dose (Figure 2B). Moreover, induction was time-dependent; as shown in Figure 2C, *EBI3* expression increased over time in response to the rDnaK, reaching a maximum at 8 h. Therefore, we conclude that *P. aeruginosa* DnaK supports the induction of *EBI3* expression.

### 2.3. DnaK-Induced EBI3 Is Involved in the Formation of IL-27

**Figure 3:**
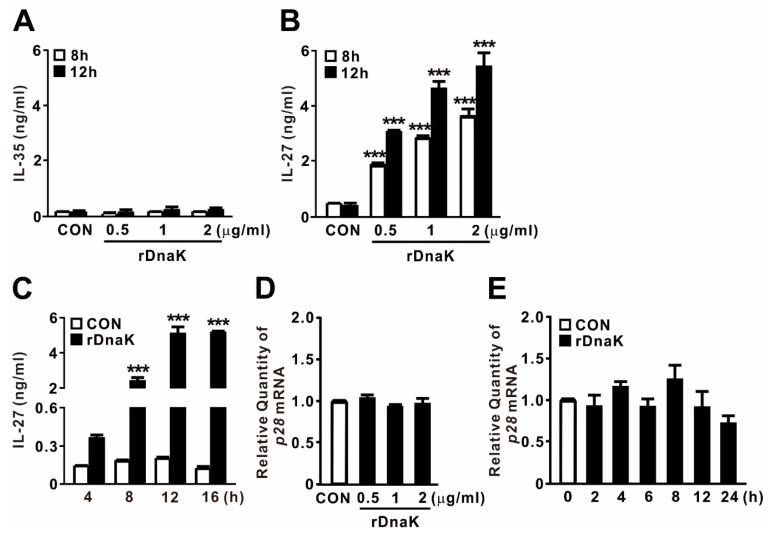
DnaK-induced EBI3 is involved in formation of IL-27. (**A**,**B**) dTHP-1 cells were treated with rDnaK at the indicated concentrations for the indicated times. (**C**) dTHP-1 cells were treated with 0.5 μg/mL rDnaK for the indicated times. (**D**,**E**) dTHP-1 cells were treated with the indicated concentrations of rDnaK for 4 h (**D**) and 0.5 μg/mL rDnaK for the indicated times (**E**). After treatment, protein levels of IL-35 and IL-27 released from dTHP-1 cells were measured by ELISA (**A**–**C**), and the *p28* mRNA level was quantified by qRT-PCR (**D**,**E**). Data are expressed as means ± SD (*n* = 3). ***, *p* < 0.001 vs. CON.

EBI3 is a structural component that forms a heterodimer with p28 or p35 to form IL-27 or IL-35, respectively. To determine whether DnaK-stimulated expression of EBI3 is associated with production of cytokines, and if so, which ones, we collected culture supernatants after treating cells with rDnaK for 8 and 12 h, and then evaluated cytokine secretion by ELISA. As shown in Figure 3A,B, we did not detect release of IL-35 at the doses tested, but we clearly observed production of IL-27 in an rDanK dose-dependent manner. In addition, when cells were exposed to 0.5 μg/mL rDnaK, release of IL-27 gradually increased over time, reaching a maximum at 12 h (Figure 3C). Given that EBI3 can form a heterodimer with the IL-27 p28 subunit to form IL-27, we asked whether expression of *p28* was also induced by the treatment with rDnaK. However, as shown in Figure 3D,E, no increase in the expression was detected, implying that production of IL-27 relies on the increase in the abundance of EBI3. Taken together, these findings indicate that rDnaK potently stimulates IL-27 production via induction of EBI3 expression.

### 2.4. IL-27 Induced by DnaK Promotes Expression of IL10 

**Figure 4:**
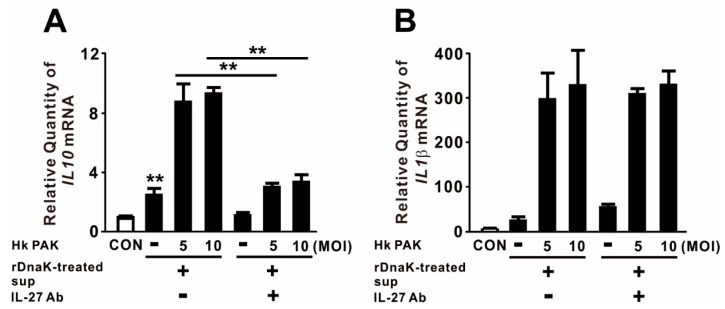
IL-27 produced in response to DnaK increases expression of *IL10*. dTHP-1 cells were treated with 1 μg/mL rDnaK for 16 h, and the supernatant was collected as conditioned medium (rDnaK-treated Sup). Conditioned media were incubated in the presence and absence of IL-27 antibody for 1 h at room temperature. These conditioned media were transferred to newly seeded dTHP-1 cells, and then the cells were treated with Hk PAK at an MOI of 5 or 10 for 4 h. After treatment, the mRNA levels of *IL10* (**A**) and *IL1β* (**B**) were measured by qRT-PCR. Data are expressed as means ± SD (*n* = 3). **, *p* < 0.01 vs. in the absence of IL-27 antibody.

Given that IL-27 is involved in a negative feedback mechanism that downregulates proinflammatory immune responses, we measured the levels of anti-inflammatory and proinflammatory cytokines in macrophages to obtain insight into the biological function of released IL-27. For this purpose, we treated dTHP-1 cells with rDnaK for 16 h and collected the supernatant as conditioned media. The conditioned media, which contained released IL-27, were transferred onto newly seeded dTHP-1 cells. These cells were treated with heat-killed (Hk) PAK at an MOI of 5 or 10 for 4 h. As shown in Figure 4A, treatment with conditioned media alone slightly increased expression of *IL10*, and expression was markedly increased by combined treatment with Hk PAK. To determine whether the increase in *IL10* expression was mediated by the induced IL-27, we neutralized IL-27 by preincubating conditioned media with an IL-27-specific monoclonal antibody before treating cells with Hk PAK. Hk PAK-mediated expression of *IL10* was decreased by preincubation of conditioned media with IL-27 antibody (Figure 4A), indicating that IL-27 contributed to cytokine expression. By contrast, Hk PAK-mediated expression of *IL1β* was not decreased by preincubation of conditioned media with IL-27 antibody (Figure 4B). Therefore, we conclude that the rDnaK-mediated release of IL-27 has a biological function.

### 2.5. DnaK-Induced Expression of EBI3 Is under the Control of the NF-κB and Akt Signaling Pathways 

**Figure 5:**
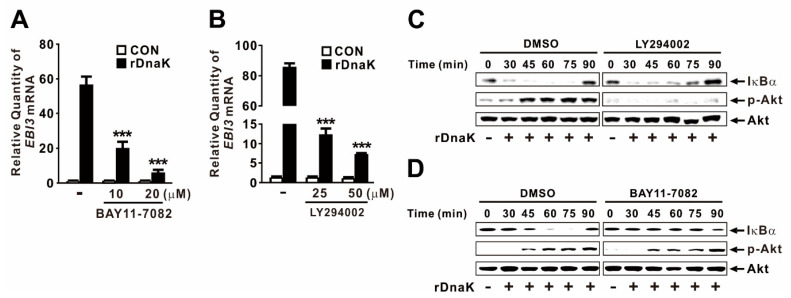
DnaK-induced expression of *EBI3* is under the control of the NF-κB and Akt signaling pathways. (**A**,**B**) dTHP-1 cells were pretreated with either BAY11-7082 (**A**) or LY294002 (**B**) at the indicated concentrations for 1 h followed by the treatment with rDnaK (1 μg/mL) for 4 h. (**C**,**D**) dTHP-1 cells were pretreated with either 50 μM LY294002 (**C**) or 20 μM BAY11-7082 (**D**), followed by treatment with rDnaK (1 μg/mL) for the indicated times. After treatment, mRNA levels of *EBI3* were measured by qRT-PCR (**A**,**B**), and protein levels were analyzed by immunoblotting (**C**,**D**). Data in **A**-**B** are expressed as means ±SD (*n* = 3). Data in (**C**,**D**) are representative of three separate experiments. ***, *p* < 0.001 vs. no treatment with inhibitor (**A**,**B**).

Akt and NF-κB are key signaling molecules involved in activation of innate immune responses to infections [41]. To determine whether rDnaK induces expression of *EBI3* through activation of NF-κB, we treated dTHP-1 cells with BAY11-7082, a chemical inhibitor of NF-κB activation, prior to treatment with rDnaK. Expression of *EBI3* was significantly decreased in a dose-dependent manner by pretreatment with the inhibitor (Figure 5A), suggesting that NF-κB is involved in the induction of rDnaK-mediated *EBI3* expression. Next, we investigated the regulatory effect of Akt by pretreating cells with LY294002, a chemical inhibitor of Akt activation, prior to treatment with rDnaK. As expected, expression of *EBI3* was also significantly decreased by pretreatment with LY294002, again in a dose-dependent manner (Figure 5B), suggesting that Akt is involved in the signaling. We verified the activation of NF-κB and Akt in response to treatment with rDnaK by monitoring degradation of IκBα and phosphorylation of Akt, respectively (Figure 5C,D). Akt engages in cross-talk with NF-κB to regulate diverse pathophysiological responses. To determine whether Akt controls the transcriptional activity of NF-κB, we pretreated cells with LY294002 prior to treatment with rDnaK. However, we barely observed a reduction in IκBα degradation (Figure 5C), indicating that NF-κB activation is not under the control of Akt. Next, to determine whether NF-κB controls the activation of Akt by inducing its phosphorylation, we pretreated cells with BAY11-7082 prior to treatment with rDnaK. As shown in Figure 5D, we did not observe any reduction in phosphorylation, indicating that Akt activation is not under the control of NF-κB. Taken together, these results suggest that rDnaK induces expression of *EBI3* via independent signaling pathways, NF-κB and Akt.

### 2.6. DnaK-Mediated Induction of EBI3 Expression Is Partly under the Control of TLR4

**Figure 6:**
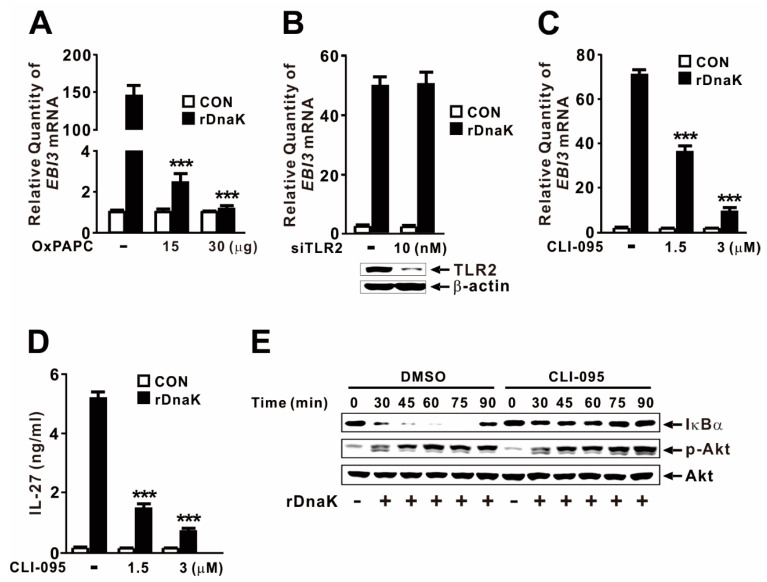
DnaK-mediated induction of *EBI3* is partly under the control of TLR4. (**A**) dTHP-1 cells were pretreated with OxPAPC at the indicated concentrations for 1 h, followed by treatment with rDnaK (1 μg/mL) for 4 h. (**B**) dTHP-1 cells were transfected with 10 nM TLR2 siRNA (siTLR2). Twenty-four hours post-transfection, the transfected cells were treated with rDnaK (1 μg/mL) for 4 h. The effect of siRNA was verified by immunoblotting for TLR2 protein. (**C**,**D**) dTHP-1 cells were pretreated with CLI-095 at the indicated concentrations for 1 h, followed by treatment with rDnaK (1 μg/mL) for 4 h. (**E**) dTHP-1 cells were pretreated with 3 μM CLI-095, followed by treatment with rDnaK (1 μg/mL) for the indicated times. After treatment, mRNA levels of *EBI3* were measured by qRT-PCR (**A**–**C**), and protein levels were measured by ELISA (**D**) or immunoblotting (**E**). Data in (**A**–**D**) are expressed as means ± SD (*n* = 3). Data in (**E**) are representative of three separate experiments. ***, *p* < 0.001 vs. no treatment with inhibitor.

Given that macrophages express multiple HSP70 receptors, including TLR2 and TLR4 [42,43], we considered it likely that DnaK induces *EBI3* expression via TLR signaling. To investigate the involvement of the TLR pathway, we pretreated dTHP-1 cells with OxPAPC, which acts as an inhibitor of TLR2 and TLR4. As shown in Figure 6A, cells pretreated with inhibitor did not exhibit induction in response to rDnaK, indicating that induction of *EBI3* expression is under the control of the TLR2 or TLR4 pathway. When we used siRNA to inhibit TLR2, DnaK-mediated *EBI3* expression was not suppressed (Figure 6B), indicating that TLR2 is not involved in the induction of *EBI3*. The siRNA-mediated reduction in TLR2 was verified by immunoblot analysis. Next, we pretreated dTHP-1 cells with CLI-095, a specific inhibitor of TLR4. As shown in Figure 6C, cells pretreated with the inhibitor expressed significantly lower levels of *EBI3* in response to rDnaK, indicating that induction of *EBI3* is under the control of the TLR4 pathway. This observation was further confirmed by measuring release of IL-27 (Figure 6D). To determine whether activation of NF-κB and Akt is under the control of TLR4, we pretreated cells with CLI-095 prior to treatment with rDnaK. Interestingly, we observed a reduction in IκBα degradation, but no change in Akt phosphorylation (Figure 6E), indicating that DnaK-initiated TLR4 signaling leads to activation of NF-κB, but not Akt. Together, these results suggest that rDnaK induces the expression of *EBI3* via NF-κB in a TLR4-dependent manner, and via the Akt pathway in a TLR4-independent manner.

## 3. Discussion

The host immune system has a broad range of defense mechanisms against infections that restrict disease progression. *P. aeruginosa* is associated with a number of PAMPs, including released proteins such as HSPs, which contribute to the induction of host immune responses to this pathogen. Consistent with this, we reported previously that *P. aeruginosa*-derived GroEL, an HSP60-like protein, stimulates an inflammatory response via PTX3 induction via NF-κB activation [44]. In addition, *P. aeruginosa* HtpG, an HSP90 homolog, upregulates IL-8 production, and this increase in expression is controlled by the NF-κB/p38 MAP kinase signaling pathways and the cylindromatosis protein [45]. However, invading pathogens have evolved to evade host immunity by downregulating host defense responses, thereby promoting pathogenesis. Neutralization of IL-27 significantly improves survival of septic mice and clearance of *P. aeruginosa*, implying that it plays a pathophysiological role during infection [16]. An important finding in this study was the observation that *P. aeruginosa* DnaK, an HSP70-like protein, potently induces the expression of *EBI3*, resulting in elevated production of IL-27, an anti-inflammatory cytokine that attenuates proinflammatory immune responses. This effect could be due to IL-27-mediated induction of IL-10 in macrophages [12,46,47], and elevated IL-10 has been implicated in susceptibility to secondary nosocomial infections in sepsis [13]. Thereby, our findings have important implications for the pathogenesis of *P. aeruginosa*, and for the interaction between this pathogen and host immunity.

DnaK is required for diverse cytoplasmic processes such as proper folding of ExoS, and thus affects the function of the T3SS apparatus [48]. DnaK is also associated with bacterial motility and adherence, as well as secretion of toxins such as elastase and exotoxin A [49]. In addition to its cytoplasmic effects, *P. aeruginosa* DnaK forms a complex with the flagella structural protein FliC and nitrite reductase NirS in the periplasm [27], and has been identified as an extracellular protein [28]. We observed an increase in *EBI3* expression in response to treatment with rDnaK. One explanation for this might be a stimulatory effect of contaminating residual endotoxin in our preparation of rDnaK. To eliminate this possibility, our rDnaK preparation technique included pretreatment with Triton X-114, which eliminates residual lipopolysaccharide (LPS) in purified proteins. In addition, we verified the absence of LPS via a limulus amoebocyte lysate (LAL) endotoxin assay, which confirmed that pretreatment reduced the contamination to <0.5 ng/mL, which was insufficient to induce *EBI3* expression under our experimental conditions. LPS-free rDnaK still clearly induced *EBI3* expression in a dose- and time-dependent manner (Figure 2B,C). Moreover, proteinase K treatment eliminated the inducing effects of rDnaK, confirming that DnaK plays a role in induction (Figure 2A). We recently reported a regulatory effect of rDnaK prepared by the same purification procedure in the control of innate immune responses [32,33].

DnaK-mediated expression of *EBI3* relies on Akt and NF-κB activation, as demonstrated by the use of chemical inhibitors such as LY294002 or BAY11-7082, as well as by monitoring phosphorylation of Akt and degradation of IκBα (Figure 5). *Mycobacterium tuberculosis* HSP70 is also involved in inducing NF-κΒ activation in human endothelial cells and IL-6 and TNF-α production in murine macrophages [50]. In addition, we used chemical inhibitors to investigate whether MAP kinases (JNK, ERK, and p38) are involved in this signaling pathway. However, expression was not mediated by these MAP kinases (data not shown). As a DAMP, HSP70 acts as a ligand in macrophages by engaging signaling receptors such as TLR2 and TLR4 [42,43]. Additionally, *EBI3* transcription is induced by surface receptors (TLR2, TLR4) and endosome receptor (TLR9) signaling in dendritic cells via NF-κB activation [51]. However, we identified TLR4, but not TLR2, as the DnaK recognition receptor that controls the induction of *EBI3* expression. Consistent with this, *Toxoplasma gondii* HSP70 induces phenotypic maturation and IL-12 production in DCs through a TLR4-dependent pathway [52]. TLR4 can sense diverse extracellular molecules, including bacterial outer membrane molecule LPS [53]. This versatility suggests that the interaction mechanisms underlying TLR4 signaling are quite diverse and may be more complicated than previously believed. In addition, monocytes and macrophages express two HSP70 receptors, Siglec-5 and Siglec-14 [54]. Given that Siglec receptors are specific to human HSP70 and do not recognize *Escherichia coli* DnaK [54], we postulated that the receptor may not be involved in recognition of *P. aeruginosa* DnaK. In support of this idea, we found that siRNA specific to Siglec-5 and Siglec-14 did not affect the extent to which *EBI3* expression was stimulated by treatment with rDnaK (data not shown). In addition, CD91 acts as a receptor for HSP70-induced innate responses in antigen-presenting cells (APCs) [55]. However, siRNA specific to CD91 did not affect the extent to which *EBI3* expression was stimulated by rDnaK treatment (data not shown).

## 4. Materials and Methods

Reagents: Proteinase K was purchased from Thermo Fisher Scientific (Waltham, MA, USA). LY294002 was purchased from Cell Signaling Technology (Danvers, MA, USA). BAY11-7082, OxPAPC, and CLI-095 were purchased from Invivogen (San Diego, CA, USA).

### 4.1. Bacterial Strains and Culture Conditions

*P. aeruginosa* wild-type strains (PAK and PAO1) [56,57] were grown on Luria (L) agar or in L broth rich medium (yeast extract, 0.5%; tryptone, 1%; NaCl, 1%; all *w*/*v*) at 37 °C. To prepare live bacteria, bacterial cells were harvested by centrifugation at 10,000× *g* for 20 min at 4 °C after overnight growth in L broth, and the bacterial pellet was resuspended in phosphate-buffered saline. To obtain culture supernatant (Sup), bacterial cells were harvested after overnight growth in minimal medium A broth (K_2_HPO_4_ 1.05%; KH_2_PO_4_ 0.45%; (NH_4_)_2_SO_4_ 0.1%; sodium citrate•2H_2_O 0.05%; glutamate 0.845%; glycerol 1%; all *w/v* except glycerol, *v*/*v*) at 37 °C [58]. The Sup was filtered through either a low protein-binding membrane with a 0.22 μm pore size (Sartorius, Goettingen, Germany) for complete removal of bacteria, or a low protein-binding 50 kDa pore size Unltracel-50 membrane (EMD Millipore, Darmstadt, Germany) for size fractionation. Hk PAK was generated by heating at 95 °C for 10 min.

### 4.2. Cell Culture

All media described below were supplemented with 10% heat-inactivated fetal bovine serum (FBS; Access, Vista, CA, USA), penicillin (100 units/mL) and streptomycin (0.1 mg/mL). THP-1 (human monocyte) cells were cultured in Roswell Park Memorial Institute (RPMI) 1640 (HyClone, Rockford, IL, USA). THP-1 cells were differentiated by incubation with 100 ng/mL phorbol 12-myristate 13-acetate for 16 h, and the resultant cells were designated as dTHP-1 cells. Cells were maintained at 37 °C in a humidified 5% CO_2_ air-jacketed incubator. Unless otherwise indicated, dTHP-1 cells were exposed to rDnaK protein at 1 μg/mL for 4 h.

### 4.3. Construction and Purification of rDnaK

Recombinant proteins were constructed and purified as described previously [32]. Phase-separation treatment with Triton X-114 was performed to remove contaminating endotoxins as described previously [59,60]; the Triton X-114 was removed using Bio-Beads SM-2 adsorbents (Bio-Rad, Hercules, CA, USA). The concentration of remaining endotoxins was <0.5 ng/mL, as determined by the LAL Chromogenic Endotoxin Quantitation Kit (Pierce Thermo, Rockford, IL, USA). Protein concentration was quantified using the BCA Protein assay kit (Pierce Thermo), adjusted to 500 μg/mL, and stored at −80 °C. To obtain control extract, *E. coli* strain BL21 (DE3) harboring a pETDuet-1 vector was subjected to the same procedures. The control extract was used to evaluate the effect of rDnaK throughout the study.

### 4.4. Quantitative Real-Time PCR (qRT-PCR)

Total RNA was isolated using TRIzol (Invitrogen, Carlsbad, CA, USA). cDNA was synthesized from total RNA using a ReverTra Ace qRT-PCR kit (Toyobo, Osaka, Japan). SYBR Green PCR Master Mix (KAPA Biosystems, Woburn, MA, USA) was used for qRT-PCR. Primer sequences were as follows: human *EBI3*, 5′-GCTCCCTACGTGCTCAATGTC-3′ and 5′-GGGCTTGATGATGTGCTCTGT-3′; human *IL1β*, 5′-AAACAGATGAAGTGCTCCTTCCAGG-3′ and 5′-TGGAGAACACCACTTGTTGCTCCA-3′; human *IL10*, 5′-GCCTAACATGCTTCGAGATC-3′ and 5′-TGATGTCTGGGTCTTGGTTC-3′; human *p28*, 5′-GAGGGAGTTCACAGTCAGC-3′ and 5′-GGTCAGGGAAACATCAGGG-3′. Reactions were performed on a CFX96 Real-Time PCR System (Bio-Rad) using the following conditions: stage 1, 50 °C for 2 min and 95 °C for 10 min; stage 2, 95 °C for 15 s and 60 °C for 1 min. Stage 2 was repeated for 40 cycles. Relative quantities of mRNA were calculated using the comparative C_T_ method and normalized against the corresponding levels of glyceraldehyde 3-phosphate dehydrogenase (GAPDH) mRNA. Primer sequences for human *GAPDH* were 5′-CCCTCCAAAATCAAGTGG-3′ and 5′-CCATCCACAGTCTTCTGG-3′.

### 4.5. Immunoblotting Analysis

Cells were collected and lysed on ice for 10 min in 20 mM Tris-HCl (pH 7.4), 50 mM NaCl, 50 mM Na pyrophosphate, 30 mM NaF, 5 μM zinc chloride, 2 mM iodoacetic acid and 1% Triton X-100 in distilled water supplemented with 1 mM phenylmethylsulfonyl fluoride (PMSF; Thermo Scientific) and 0.1 mM sodium orthovanadate (Sigma-Aldrich, St. Louis, MO, USA). Lysates were centrifuged at 10,000× *g* for 15 min at 4 °C, and protein concentration was measured using the bicinchoninic acid method (BCA; Pierce Thermo). Proteins were separated by 10% SDS-PAGE and transferred to 0.45 μm polyvinylidene difluoride membranes. Membranes were blocked in tris-buffered saline (TBS; 10 mM Tris-HCl (pH 7.5), 150 mM NaCl) with 5% nonfat dry milk solution at room temperature for 1 h, and then incubated for 16 h at 4 °C with primary antibodies against IκBα, p-Akt, Akt, TLR2 (D7G9Z), and β-actin (Cell Signaling Technology). After washing, the membranes were incubated with the corresponding horseradish peroxidase (HRP)-conjugated secondary antibodies for 1 h at room temperature. Protein bands were visualized using an ImageQuant LAS-4000 system (GE Healthcare Life Sciences, Chicago, IL, USA) following addition of WEST-ZOL plus Chemiluminescent Substrate (Intron, Seongnam, South Korea).

### 4.6. Enzyme-Linked Immunosorbent Assay (ELISA)

The amounts of IL-27 and IL-35 released into supernatants were measured using the Human IL-27 Duoset ELISA kit (R&D Systems, Minneapolis, MN, USA) and Human EBI3/IL27B ELISA kit (LifeSpan Biosciences, Seattle, WA, USA).

### 4.7. Transfection of siRNA

Cells (9 × 10^5^/mL) were seeded into 12-well tissue culture plates and transfected with the recommended concentrations of siRNA targeting *TLR2* (siTLR2, #sc-40256; Santa Cruz Biotechnology, Dallas, TX, USA) using Lipofectamine RNAi Max (Invitrogen). Transfected cells were incubated for 48 h at 37 °C in RPMI 1640 supplemented with 10% FBS. Transfection efficiency was assessed using cells transfected with fluorescein amidites (FAM)-labeled mimics. Total RNA and protein were harvested for qRT-PCR and immunoblot analysis, respectively.

### 4.8. Statistical Analysis

Statistical analyses were performed with Student’s *t*-test or one-way ANOVA followed by Tukey’s post-hoc multiple range test. Calculations were performed using the Instat package from GraphPad Software (San Diego, CA, USA). A *p*-value < 0.01 was considered statistically significant.

## 5. Conclusions

Our findings reveal that *P. aeruginosa* DnaK potently stimulates *EBI3* expression, resulting in upregulation of IL-27 production in macrophages. As a non-self-antigen released by *P. aeruginosa*, DnaK functions as a PAMP to restrict host immune responses by increasing the production of IL-10. In human macrophages, the effects of DnaK are primarily associated with recognition by TLR4 and subsequent signal transduction via NF-κB. In addition, these effects are also under the control of Akt, which is activated by unidentified receptors. Future studies should seek to identify the receptors involved in DnaK-mediated activation of Akt. Such studies would contribute to our understanding of the diseases caused by *P. aeruginosa* infection and provide new opportunities for their treatment.
